# Effect of Bilingualism on Lexical Stress Pattern Discrimination in French-Learning Infants

**DOI:** 10.1371/journal.pone.0030843

**Published:** 2012-02-17

**Authors:** Ranka Bijeljac-Babic, Josette Serres, Barbara Höhle, Thierry Nazzi

**Affiliations:** 1 Université Paris Descartes, Paris, France; 2 CNRS, Laboratoire Psychologie de la Perception, Paris, France; 3 Université de Poitiers, Poitiers, France; 4 Universität Potsdam, Potsdam, Germany; University of Barcelona, Spain

## Abstract

Monolingual infants start learning the prosodic properties of their native language around 6 to 9 months of age, a fact marked by the development of preferences for predominant prosodic patterns and a decrease in sensitivity to non-native prosodic properties. The present study evaluates the effects of bilingual acquisition on speech perception by exploring how stress pattern perception may differ in French-learning 10-month-olds raised in bilingual as opposed to monolingual environments. Experiment 1 shows that monolinguals can discriminate stress patterns following a long familiarization to one of two patterns, but not after a short familiarization. In Experiment 2, two subgroups of bilingual infants growing up learning both French and another language (varying across infants) in which stress is used lexically were tested under the more difficult short familiarization condition: one with balanced input, and one receiving more input in the language other than French. Discrimination was clearly found for the other-language-dominant subgroup, establishing heightened sensitivity to stress pattern contrasts in these bilinguals as compared to monolinguals. However, the balanced bilinguals' performance was not better than that of monolinguals, establishing an effect of the relative balance of the language input. This pattern of results is compatible with the proposal that sensitivity to prosodic contrasts is maintained or enhanced in a bilingual population compared to a monolingual population in which these contrasts are non-native, provided that this dimension is used in one of the two languages in acquisition, and that infants receive enough input from that language.

## Introduction

Growing up in a bilingual environment is a reality for the vast majority of children. Children acquiring more than one language simultaneously are exposed to a more complex and less homogenous speech input than monolinguals. Recently [1] suggested that there are not only quantitative but also qualitative differences in the input received by bilinguals compared to monolinguals (since both languages can be produced by native or non-native speakers) which could affect the acquisition process. Moreover, given that languages differ in segmental properties, prosody, lexicon, and syntax, it is believed that bilinguals' language processing abilities will necessarily differ from those of monolinguals, and will differ amongst bilinguals according to the specific combination of languages they are acquiring. Nevertheless, bilingual infants and children generally succeed in simultaneously learning their two languages, with some evidence that they are going through the language development milestones at the same ages as their monolingual peers [2]. Hence, language acquisition in bilinguals might not be as challenging as it seems, and it might actually rely on the remarkable plasticity of early speech processing systems, as found in monolingual infants' ability to process information in foreign languages [3]–[4] in spite of their early specialization for the processing of the native language [5]–[7].

In the present study, we evaluate how bilingual acquisition might affect how French-learning infants raised in bilingual as opposed to monolingual environments perceive stress pattern contrasts. Before describing the study in more detail, we first review what we know about the speech perception abilities of bilingual infants exposed from birth to two different languages. So far, most studies have been conducted on language discrimination and phonetic perception, and present evidence suggests that bilingual infants' speech perception abilities develop in the first months of life at the same pace as in monolingual populations.

First, language discrimination abilities appear to develop in a parallel way in bilingual [8]–[9] and monolingual [10] infants. In fact, the ability of both newborn and 2-month-old monolingual infants to discriminate languages if they differ by their overall rhythmic properties but not if they have similar rhythms [11]–[13] has recently been extended to bilingual newborns [14]. Moreover, following evidence of discrimination of languages of the same rhythmic class by 4 months of age in monolinguals [8]–[10] bilingual Catalan/Spanish 4-month-olds were found to be able to discriminate between their two languages, which belong to the same rhythmic class [9], and between one of their languages and a foreign language of the same rhythmic class [8]. The only difference found between monolingual and bilingual 4-month-olds was that bilinguals orient faster to a foreign language than to one of their native languages, while monolinguals show the opposite pattern [8]. Overall, these results suggest similar refinements in language discrimination abilities over the course of early development in monolingual and bilingual infants. However, due to the nature of the stimuli used in these studies (relatively complex sentences, containing rich phonetic and prosodic information), these developmental changes have been given two interpretations. They have been taken as possible signs of the acquisition of some specific prosodic properties of the native language, such as the way utterance boundaries are marked or stress is acoustically realized [9]–[10]. Alternatively, it has been proposed that infants might react to segmental differences in the exact inventory and acoustic realizations of phonemes in the languages being discriminated [9]. Both monolingual and bilingual infants may have acquired these prosodic and segmental properties of the native language in similar ways. In order to better understand what properties these infants have acquired, one needs to work with more controlled stimuli. In the following, we review studies that have begun the process of mapping segmental acquisition in bilingual infants. Because they give us insights into phonological acquisition in bilingual infants, they are important for framing the present study, the first to explore the acquisition of prosody in bilingual infants.

Bosch and Sebastian-Galles [15], who used a familiarization-preference procedure to test bilingual infants learning both Spanish and Catalan, conducted the first study that evaluated the acquisition of segmental contrasts in bilingual infants and monolingual Catalan and Spanish infants on their discrimination of a Catalan-specific vowel contrast (/e/–/E/) that is not present in Spanish. At 4 months of age, the two monolingual populations and the bilinguals were able to discriminate the contrast. Discrimination abilities were not found at 8 and 12 months in the Spanish monolinguals, while they were maintained in the Catalan monolinguals, a pattern that is predicted by previous studies on phonetic category acquisition in monolinguals that show a reduction in discrimination of non-native phonetic contrasts by the end of the first year of life [5], [16]. In contrast to the Catalan monolinguals, discrimination of this vowel contrast was not found in the Spanish-Catalan bilinguals at 8 months, while evidence of such discrimination was again found at 12 months. This U-shaped acquisition pattern in this bilingual population was generalized to another phonetic contrast specific to the Catalan language (/s/ vs. /z/) [17], and also to a phonetically close contrast present in both Spanish and Catalan (/o–u/), though not to a more distant one present in both languages (/e–u/) [18].

This U-shaped pattern was initially explained by phonological changes that occur around 8 months, when infants acquire the inventories of phonetic categories of their native language(s). However, these transitory difficulties in discriminating some phonetic contrasts were not replicated using a different method, the anticipatory eye movement paradigm. Albareda-Castellot and colleagues [19] evaluated the discrimination abilities of Catalan-Spanish bilingual and respective monolingual 8-month-old infants for both a Catalan-Spanish vowel contrast, and the same Catalan-specific vowel contrast as used in [15]. Unlike the results with the original procedure, the new results established discrimination of the Catalan-specific contrasts by the bilingual 8-month-olds. Even if these results require further confirmation, they suggest that the acquisition of language-specific phonetic categories in monolingual and bilingual infants is similar and that bilinguals are sensitive to the contrasts of the two languages throughout the first year of life, which can be demonstrated when using appropriate tasks (see also [18]). This interpretation is further supported by findings from bilinguals learning another pair of languages (English and French). Studies exploring the discrimination of a French [b] – [p] contrast and an English [b] – [p^h^] contrast [20], or discrimination between dental (French) and alveolar (English) variants of the [d] phoneme [21] have established that performance is predicted by infants' linguistic environment both in monolinguals and in French-English bilinguals. Therefore, it appears that the time course of segmental acquisition is similar in bilingual and monolingual populations (although slight, fine-grained differences might exist, possibly due to differences in attention abilities, which might also explain the slight differences found for language discrimination abilities).

While the above studies are beginning to provide insight into early segmental acquisition in bilingual infants, nothing is known regarding early prosodic acquisition in bilingual infants, even though production studies suggest differences between prosodic acquisition in monolingual and bilingual children aged 2;6–6;0 years [22]–[24]. However, because later production differences need not originate from early differences in perception, the present study was designed to evaluate prosodic perception in the first year of life. More specifically, we aimed to investigate bilinguals' ability to discriminate lexical stress patterns (strong-weak versus weak-strong), testing 10-month-old bilinguals learning both French and a language with lexical stress.

This question is important for the following reasons. First, the age of 10 months was chosen because, while infants are sensitive to stress patterns at birth [25]–[27], effects of the prosodic structure of the native language on perception have been found to emerge between 5 and 10 months [28]–[33], and to have consequences on speech processing [34]–[36]. Hence, by 10 months, prosodic perception has become language specific and bilinguals would potentially have to cope with two different systems. Second, the languages of the infants were chosen so that the infants would learn two languages that differ radically in their use of stress at the lexical level. As one of their languages, all infants were learning French, which, contrary to many languages, does not use stress contrasts at the lexical level, and has actually very little lexical accentuation, since what lexical accentuation it has is marked by a lengthening of the last syllable of only phrase-final words [37]. The other language they were learning (differing across the infants) was one that uses stress at the lexical level. Thus, these infants would need to learn two prosodic systems and would have to process lexical prosody differently in their two languages. Based on studies showing crosslinguistic differences in the processing of lexical prosody in adults and infants learning French versus a language with lexical stress such as Spanish or German, we hypothesized that they would be more sensitive to stress contrasts than French-learning monolinguals of the same age, and that this increase might be modulated by the amount of input of the stress-contrasted language and/or by the balance between their two languages.

Regarding adults, several studies have found that native speakers of French have more difficulties in perceiving lexical stress than native speakers of Spanish, a phenomenon sometimes called stress perception “deafness”, even though stress pattern discrimination is above chance level in French adults, at least in the easiest experimental conditions [38]–[39]. Furthermore, the perception of lexical stress by simultaneous French-Spanish bilinguals shows intermediate performance between that of Spanish native speakers and that of French late learners of Spanish. When separating the bilinguals according to their dominance, the performance of the Spanish-dominant bilinguals was similar to that of Spanish monolinguals, but the performance of the French-dominant bilinguals was very similar to that of French late learners of Spanish [40]–[41]. In other words, these results highlight the strong impact of language dominance on stress pattern processing even in simultaneous and very fluent adult bilinguals. Therefore, they raise the question of the importance of the balance between the two languages of a bilingual in the development of his or her language-specific prosodic abilities, an issue that, in the present study, will be addressed in infants.

Regarding infants, three recent studies have compared stress pattern discrimination in French- versus Spanish- [33] or German-learning [30]–[31] infants. First, French-learning infants appear to discriminate trochaic versus iambic stress patterns by 4-to-6 months of age, as found at 4 months using ERPs and a single token of each stress pattern (/baba/) [30] and at 6 months using the headturn preference procedure (HPP) as a discrimination technique (test preceded by a 1-minute familiarization phase with one of the two stress patterns) and several exemplars of the same disyllabic sequence (/gaba/) for each stress pattern [31]. Moreover, in both studies, crosslinguistic differences were found. In the ERP study, the pattern of discrimination differed for the French- and German-learning infants, suggesting that at the neuronal level, stress information is already processed in a language-specific way at 4 months. In the HPP study, discrimination at 6 months was accompanied by a preference for the trochaic pattern in the German-learning infants, while discrimination but no preference for either of the stress patterns was observed in the French-learning infants.

A few months later, crosslinguistic differences in stress pattern discrimination are still found, but now marked by lower performance in French-learning infants than in infants learning a language with lexical stress. Skoruppa and colleagues [33] tested stress discrimination in French- and Spanish-learning 9-month-olds, using HPP as a discrimination technique (with a 2-minute familiarization phase, hence twice as long as the one used in [31]). They found that if the two stress patterns are exemplified by lists of segmentally different words (8 different words in familiarization and 8 new different words at test), Spanish-learning infants can discriminate them while French-learning infants cannot. However, French-learning infants succeed at that age if presented with different tokens of a single sequence (/pima/) for each stress pattern. These data were interpreted as evidence that French infants, who are learning a language without contrastive lexical stress, do not encode stress at the abstract phonological level (i.e. when listening to a list of phonetically different words) although they are able to discriminate the stress contrast at the phonetic level (i.e. when only one phonetic sequence is presented). However, the French-learning 9-month-olds' discrimination performance was obtained after a relatively long familiarization phase of 2 minutes, so that it is possible, given the “stress deafness” data on French-speaking adults [38]–[39], that infants would have failed to discriminate with a shorter 1-minute familiarization phase as used with 6-month-olds [31]. Although this is an empirical issue and no data on this issue already exists, this possibility would be compatible with the Hunter and Ames model [42] according to which novelty preferences, as reported by Skoruppa and colleagues [33], are usually found in relatively easy discrimination conditions (while familiarity preferences are obtained in more difficult discrimination conditions, as found by Höhle and colleagues [31], with the shorter familiarization phase and the younger infants). Therefore, before evaluating in Experiment 2 the effect of learning two different prosodic systems (one with lexical stress and one without) on the perception of prosody, the present study had to further explore French-learning monolinguals' perception of lexical stress pattern differences. In particular, since we hypothesized that our targeted bilinguals would have better stress pattern discrimination performance than French-learning monolinguals, we were interested in identifying an experimental situation in which the monolinguals would fail to show evidence of discrimination (contrary to what was found in [33]) in order to then test the bilinguals with the prediction that they would show discrimination. Accordingly, on the basis of predictions derived from Hunter and Ames [42], Experiment 1 tested the stress pattern discrimination performance of 10-month-old French-learning monolingual infants, in two experimental conditions: following a 2-minute familiarization as done in [33], and following a 1-minute familiarization as done in [31]. In both cases, the stimuli were the same as those used in [31]. Our prediction was that a 1-minute familiarization would not allow these infants to discriminate the stress patterns, whereas a 2-minute familiarization would. This would extend Skoruppa and colleagues' [33] findings to new stimuli produced in a different language (German, as opposed to Spanish).

## Methods

### Participants

All participants were without apparent health problems, and had at least 37 weeks of gestation. Participants were recruited from birth-lists obtained through the Paris city hall archives, or through a database of families who had previously participated in speech perception studies in our laboratory. Informed written consent was obtained from all parents.

Experiment 1: Thirty-two French-learning monolingual infants aged 10 months (M = 10;21; range: 10;04–11;18) participated in this experiment. Seventeen additional infants were excluded due to fussiness (9), technical problems or experimenter error (8).

Experiment 2: Thirty-two bilingual 10-month-olds (M = 10;21; range: 9;27–12;12) who were learning both French and another language were tested in the short familiarization condition. Ten additional bilingual infants were excluded due to fussiness (2), technical problems (1), and failing to meet the criterion of language distribution (7). All infants had been exposed to their two languages from birth, but varied in terms of the balance of their linguistic input. The infants' language exposure, that is the amount of time people regularly interact with the infant during the week and the weekends, was measured with the Language Exposure Questionnaire [8]. Since a review of the literature revealed that the upper-limit criterion for classifying infants as bilinguals varies between studies (from 65–35%, e.g. [9], to 79–21% [43]), and since we wanted to distinguish two subgroups of bilinguals, those with a rather balanced input (subgroup 1: “balanced” bilinguals), and the others with a bias in favor of the language other than French (subgroup 2: “dominant other language” bilinguals), we decided to use the full range covered in the literature. Therefore, the 16 infants in subgroup 1 were hearing both languages 40–60% of the time, with a mean of 48.3% for the language other than French, while the 16 infants in subgroup 2 were exposed to the language other than French 70–80% of the time, with a mean of 73.4%. Note that in the present study another reason for considering an infant receiving 20% French – 80% other language as bilingual rather than monolingual is related to the fact that these numbers are mostly based on home input, and thus do not take into account exposure to French outside the home since all infants were growing up in Paris, which affects our calculation of the % of French exposure, particularly for infants growing up in languages in which the language other than French is dominant.

The bilingual infants also varied in terms of the language other than French that they heard, with 15 different languages being spoken in the bilingual infants' families (see Table 1). We verified that all of these languages use stress contrasts at the lexical level (although we could not define the acoustic factors used to mark stress in all these languages, see more on this issue in the General Discussion), based on different kinds of information provided by *The World Atlas of Language Structures Online* [44]. In that database, English, Kabyle, and Spanish are described as trochaic languages, Italian, Russian, Urdu and Swedish are described as having variable stress, Portuguese as having penultimate stress, Fulfulde as having stress-accent on the penultimate and initial positions, Hebrew as having a stress-accent on the last syllable and Serbian, Slovak and Polish as having fixed stress. Lastly, according to Angoujard [45] , word stress-accent falls on the penultimate or antepenultimate syllables in most African Arabic languages (the type of Arabic languages spoken in the homes of our participants), while according to Schiering and Bickel [46] in Vietnamese the last syllable usually receives heavy stress when several syllables are combined into di- or trisyllabic strings. Since the stimuli were produced by a German speaker, French-German bilingual infants were excluded from this group so that the stimuli were produced in a foreign language for all infants.

**Table 1:** Number of bilingual infants (“balanced” versus “dominant other language”) hearing each of the 15 languages other than French in their environment.

Language	“balanced” bilinguals	“dominant” bilinguals
Spanish	4	3
English	3	2
Portuguese	2	3
Arabic	1	1
Italian	2	0
Russian	0	2
Fulfulde	1	0
Hebrew	1	0
Kabyle	1	0
Polish	0	1
Serbian	0	1
Slovak	0	1
Swedish	0	1
Urdu	0	1
Vietnamese	1	0

### Stimuli

In both experiments the stimuli were CVCV /gaba/ sequences, stressed either on the first or on the second syllable. Several tokens of each stress pattern were recorded by a female German native speaker (for details, see [31]). The tokens were used to create 6 files for each stress pattern that differed in the order of presentation of the different tokens, the tokens in a file being separated by pauses of about 600 ms. The trochaic speech files had an average duration of 18.39 s (range: 18.28 s to 18.51 s; average duration of trochaic items: 591 ms) and the iambic files had an average duration of 18.01 s (range: 18.00 s to 18.07 s; average duration of iambic items: 603 ms).

### Procedure

The classic version of the Headturn Preference Procedure (HPP) was used [28]. Each trial began with the green light on the center panel blinking until the infant oriented to it. Then, the red light on one of the side panels began to flash. When the infant turned in that direction, the stimulus for that trial began to play. It was played to completion or stopped immediately if the infant failed to maintain the headturn for 2 consecutive seconds (if the infant turned away for less than 2 s, the trial continued but the time spent looking away was excluded). The procedure, intended to test discrimination, consisted of a familiarization phase followed by a test phase [31]. Infants were familiarized with one of the 6 trochaic or iambic files until they reached a familiarization criterion. Half of the infants in each subgroup were familiarized with the trochaic pattern, the other half were familiarized with the iambic pattern. Once the familiarization criterion was reached, infants were tested with two different files of the same stress pattern, and two files of the opposite stress pattern. This block of four files was repeated three times, with varied random presentation orders, leading to the presentation of 12 test trials, half of the same and half of the opposite stress pattern. The file used during familiarization and the four files used during test, chosen among the 6 trochaic and 6 iambic files, were counterbalanced across infants. Only the familiarization criterion varied between Experiment 1 and Experiment 2. In experiment 1, for one subgroup of monolingual infants the familiarization criterion was set to 2 minutes of orientation time (long familiarization) and to 1 minute (short familiarization) for the other subgroup. In Experiment 2 all bilingual participants were familiarized during only 1 minute (short familiarization).

## Results

Experiment 1: As in previous studies [31], we checked for individual orientation times exceeding 18 s to control for potential effects of the slightly longer duration of the trochaic stimuli. Three trials were reduced to 18 s, accounting for 1% of all trials.

**Figure 1:**
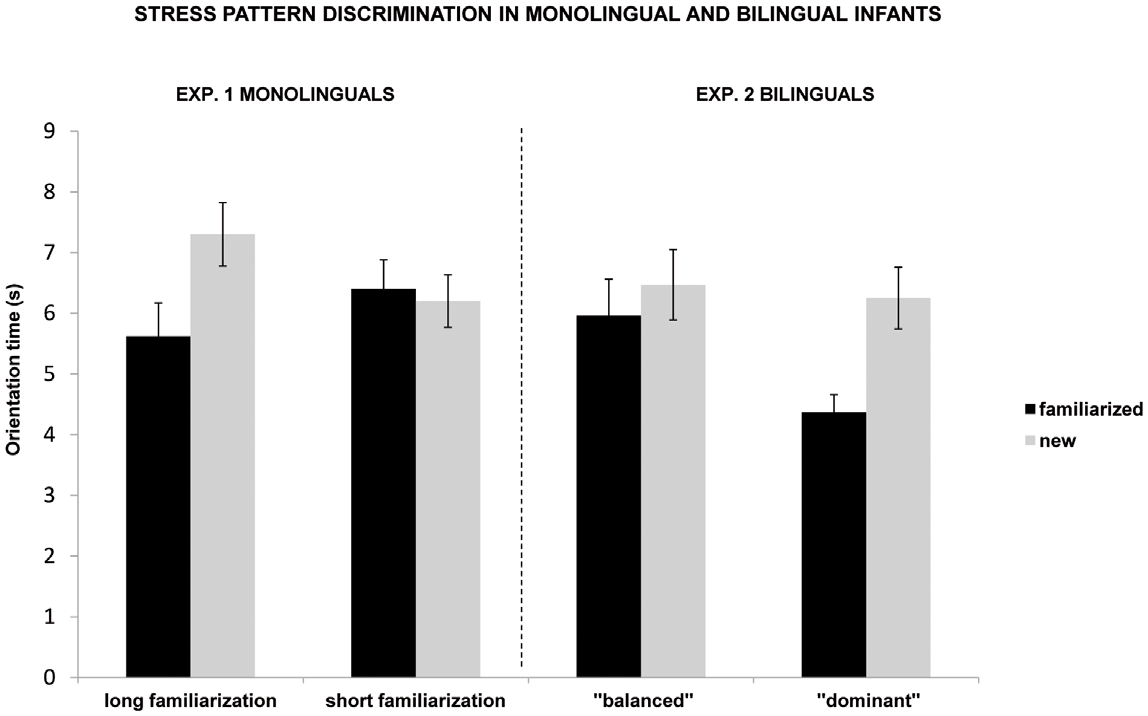
Stress pattern discrimination in monolingual and bilingual infants. Mean orientation times to the familiarized and new stress patterns for 10-month-old infants. Experiment 1: monolinguals in short and long familiarization conditions. Experiment 2: bilinguals (“balanced” and “dominant other language”) in short familiarization condition. The error bars indicate the standard error of the mean.

An ANOVA with the within-subject factor of familiarity (familiarized versus new stress pattern) and the between-subject factors of pattern (familiarization with trochaic versus familiarization with iambic pattern) and condition (short versus long familiarization) was conducted. It revealed a significant main effect of familiarity, F(1, 28) = 7.16, p = .012, ηp^2^ = .20, and a significant interaction between condition and familiarity, F(1, 28) = 11.39, p = .002, ηp^2^ = .29. This interaction is due to the fact that the subgroup of French monolingual infants in the long (2-minute) familiarization condition oriented less to the sequences with the familiarized stress pattern (M = 5.62 s; SE = .55) than to the sequences with the new stress pattern (M = 7.30 s; SE = .52), F(1, 28) = 18.3, p = .0002, ηp^2^ = .39. Twelve out of 16 infants had longer orientation times to the new stress pattern (p = .038, binomial test). In contrast, French monolinguals in the short (1-minute) familiarization condition oriented equally to the sequences with the familiarized stress pattern (M = 6.44 s; SE = .48) and to the sequences with the new stress pattern (M = 6.24 s; SE = .43), F(1,28)<1. Six out of 16 infants had longer orientation times to the new stress pattern (p = .23, binomial test). All other effects and interactions failed to reach significance (all F(1, 28)<1). See Figure 1, left side.

The results from the long (2-minute) familiarization condition, in which a significant novelty effect was found, establish that French-learning 10-month-olds can discriminate different stress patterns at the phonetic level, a finding consistent with the data from [33] for 9-month-olds using a similar procedure but different stimuli. In this context, the null result obtained in the short (1-minute) familiarization condition cannot be due to an inability to distinguish the stress patterns *per se*, but has to be interpreted as a direct consequence of the shorter familiarization, suggesting that stress pattern discrimination is relatively hard to elicit at 10 months in French-learning monolingual infants. Note that this finding of a null result with a shorter familiarization and a novelty preference with a longer familiarization, all other things being equal, is in accordance with the predictions of Hunter & Ames [42]. With this pattern of results in hand, we can now turn to our principal aim which was to evaluate the impact of bilingual acquisition on the discrimination of lexical stress pattern in 10-month-olds who are growing up learning both French and a second language that has lexical stress.

Because we hypothesized that the presence of a stress-contrasted language would maintain/enhance the bilinguals' sensitivity to stress contrasts at a higher level than French-learning monolinguals, we tested the bilinguals in the short familiarization condition. Our rationale was that if these bilinguals are more proficient at processing stress than French-learning monolinguals, then a short familiarization should be enough to elicit discrimination, marked by a novelty effect, in the test phase for that group. The bilinguals stress-contrasted language was varied between infants (15 languages were used), corresponding to the diversity of bilingual families in the Paris area. Importantly, the use of this large spectrum of bilinguals was motivated by our assumption that it is the presence of lexical stress contrast that would be the cause of increased stress pattern discrimination for bilinguals compared to monolinguals, and that this should play a role even if the exact realization of stress and its position within the word differs from the marking present in our stimuli (recorded by a German native speaker). In addition, given Dupoux and colleagues' [40] results showing effects of the relative balance between languages on prosodic processing, two subgroups of bilinguals were constituted on the basis of time of exposure to the second language in Experiment 2. We hypothesized that if hearing a language with lexical stress maintains/enhances stress pattern discrimination, a discrimination effect would be found in these bilingual groups (subgroup 1: “balanced” bilinguals and subgroup 2: “dominant other language” bilinguals), or at least in subgroup 2. Indeed, if amount of input to relevant stress pattern information plays a role, then this effect should be stronger in subgroup 2 and performance for subgroup 1 would either be above chance level but below that for subgroup 2, or at chance level.

Experiment 2: We also checked for individual orientation times exceeding 18 s, but none were found. Results are presented in Figure 1, right side. An ANOVA with the within-subject factor of familiarity (familiarized versus new stress pattern) and the between-subject factors of pattern (familiarization with trochaic versus familiarization with iambic pattern) and language exposure (subgroup 1 vs. subgroup 2) was conducted. It revealed a significant main effect of familiarity, F(1, 28) = 21.99, p = .0001, p^2^ = .44 and a significant interaction between familiarity and language exposure, F(1, 28) = 4.25, p = .049, ηp^2^ = .13. This interaction is due to the fact that “balanced” bilingual infants from subgroup 1 tended to orient less to the sequences with the familiarized stress pattern (M = 5.83 s; SE = .60) than to the sequences with the new stress pattern (M = 6.53 s; SE = .58), but this difference was not significant, F(1, 28) = 3.43, p = .07, ηp^2^ = .11. Eleven out of 16 infants had longer orientation time to the new stress pattern (p = .105, binomial test). They were learning English (2 infants), Portuguese (2), Spanish (4), Fulfulde, Hebrew and Italian. The 5 infants with negative scores were learning Arabic, English, Italian, Kabyle and Vietnamese. In contrast, infants in subgroup 2 (the “dominant other language” bilinguals) also oriented less to the sequences with the familiarized stress pattern (M = 4.37 s; SE = .29) than to the sequences with the new stress pattern (M = 6.25 s; SE = .51), but this difference was significant, F(1, 28) = 22.97, p = .00001, ηp^2^ = .45. Twelve out of 16 infants had longer orientation times to the new stress pattern (p = .038, binomial test). They were learning English (1 infant), Portuguese (3), Spanish (3), Russian (2), Chinese, Polish and Serbian. The 4 infants with negative scores were learning Arabic, English, Slovak and Swedish. All other effects and interactions failed to reach significance (all F(1, 30)<1). Therefore, independently of the stress pattern presented during familiarization, bilingual infants showed a discrimination effect, but only if they belonged to the “dominant other language” subgroup. This difference in performance between the two subgroups of bilinguals does not seem to be related in any obvious way to which language other than French they were also hearing.

To compare bilinguals' performance in the present experiment and monolinguals' performance in the short familiarization condition in Experiment 1, a second ANOVA with the within-subject factor of familiarity (familiarized versus new stress pattern) and the between-subject factors of group (monolingual vs. bilingual subgroup 1 vs. bilingual subgroup 2) and pattern (familiarization with trochaic versus iambic pattern) was performed. It revealed a significant effect of familiarity, F(1, 42) = 12.67, p = .001, ηp^2^ = 0.23 and no effect of group, F(2, 42) = 1.40, p = .26. Importantly, there was a significant interaction between familiarity and group, F(2, 42) = 7.06, p = .002, ηp^2^ = 0.23. Planned comparisons restricted to two-by-two group comparisons revealed a larger familiarity effect for the bilinguals that were dominant for the language other than French, as attested by significant familiarity×group interactions when comparing the dominant bilinguals with either the monolinguals, F(1, 42) = 14.08, p = .0005, ηp^2^ = 0.25, or the balanced bilinguals, F(1, 42) = 4.27, p = .045, ηp^2^ = 0.09. The interaction restricted to the balanced bilinguals and the monolinguals was not significant, F(1, 42) = 2.78, p = .10, ηp^2^ = .06. All other effects and interactions failed to reach significance (all Fs<1). Therefore, French/other language bilingual 10-month-olds appear to be more sensitive to stress pattern than French-learning monolinguals of the same age but only when the quantity of exposure to the other language is much higher than the exposure to French.

It is noteworthy that because both the monolinguals and the bilinguals had never heard German spoken in their environment (French-German infants having been purposefully excluded from the present study), the behavioral differences observed between the two groups cannot be explained by differences in their familiarity with the language properties (and in particular the phonetics) of the language in which the stimuli had been recorded (German), but rather by differences in the mechanisms used in both populations to process prosodic information. In relation to this point, note that the results of Skoruppa and colleagues [33] do not exclude the possibility that part of why French-learning infants had difficulties in processing the stress patterns and performed less well then Spanish-learning infants, was due to the fact that the stimuli had been pronounced in Spanish, hence with phonetic properties that only matched the phonology of the native language of the latter, but not the former, group of infants.

## Discussion

The goal of the present study was to explore for the first time stress sensitivity at the lexical level in bilingual infants learning two languages with different lexical stress systems. To investigate this, bilingual infants were tested at 10 months given prior reports of language-specific prosodic sensitivity in monolinguals at that age [30]–[31], [33]. The targeted population corresponded to bilingual infants learning both French and another language. This bilingual population varied in two ways: the balance of their two languages (“balanced” versus “dominant in other language“), and the language other than French they were acquiring (15 different languages included). The bilinguals' sensitivity to stress (Experiment 2) was compared to that of French-learning monolingual infants of the same age and in the same 1-minute familiarization condition (Experiment 1). We had hypothesized that the bilingual infants would be more sensitive to stress contrasts than the monolinguals as a result of their learning a language with lexical stress in addition to French. We had also hypothesized that this effect would be stronger for (or maybe even restricted to) the bilinguals who are dominant in the language with lexical stress than for the more balanced bilinguals.

Experiment 1, with French-learning monolingual 10-month-olds, was run to establish a behavioral baseline to which bilinguals could be compared. The results show that these infants can discriminate the trochaic and the iambic stress patterns following a long (2-minute) familiarization to one of the two patterns, as attested by a novelty preference, but not after a short (1-minute) familiarization. From the long familiarization discrimination result, we can conclude that by 10 months of age, French-learning infants are not “deaf” to lexical stress contrasts (even though such contrasts are absent in their native language) when presented with lists of different exemplars of a single CVCV sequence. Instead, they perceive differences between stress patterns if they are presented with only one pseudo-word and if the familiarization time is long enough. This finding is congruent with the results in [33], in which French-learning monolingual 9-month-olds were found to be able to discriminate stress patterns at the phonetic level (when given lists of different tokens of the same pseudo-word, as done in our study) but not at the phonological level (when given lists of different pseudo-words). Therefore, our study extends French-learning infants' prosodic discrimination abilities to another set of speech stimuli, recorded by a German speaker rather than a Spanish speaker. However, our results go beyond those of Skoruppa and colleagues [33] in showing that discrimination can only be elicited after 2 minutes of familiarization to one pattern, not after only 1 minute, suggesting some limits in the processing of stress patterns in this population of French-learning monolingual 10-month-olds. This finding suggests that stress pattern discrimination might be relatively hard to elicit at 10 months in French-learning monolingual infants, a result confirmed by the results from the bilingual infants in Experiment 2. The direction of the effects (null result in the more difficult, shorter familiarization, and a novelty effect in the easier, longer familiarization) are in accord with the Hunter & Ames model [42].

Regarding the effect of bilingualism, the comparison of the results of our two experiments clearly establishes that tested under the same conditions (1-minute familiarization time), bilinguals learning French together with a language that has lexical stress are more sensitive to stress pattern contrasts than monolinguals, but only if they are dominant in their stress-contrasted language, not if they received a balanced input of their two languages. As mentioned earlier, this difference in performance does not seem to be due to differences in the specific languages heard by infants in the two subgroups. These findings on the effects of language balance are reminiscent of the results in [40] of an effect of language dominance in French-Spanish bilingual adults' discrimination of stress pattern, although little is known about the precise link between early language balance and later language dominance. This modulation by language balance suggests that the increased performance in the group dominant for the stress-contrasted language is not due to general cognitive effects of bilingualism - such as increased ability to learn different rules simultaneously, or increased ability to avoid interference - that are found both in adulthood [47] and infancy [48]–[49], but is instead due to the nature and quantity of the language input received.

Regarding the bilinguals dominant in the stress-contrasted language, it is important to note that increased sensitivity was found in a group with various languages that all have lexical stress, from which infants also learning German, the language in which the stress contrasts used in the present study were produced, were excluded. This suggests that this increased sensitivity can be used to discriminate stimuli produced with acoustic properties different than the ones heard by the bilingual infants in their bilingual environment. This point raises several questions to be evaluated in future research, related to whether the locus of the effect observed here is at the phonetic or the phonological level. First, given that this discrimination effect was found in 12 of the 16 infants tested, who heard 7 of the 10 languages heard by infants in this group, it is plausible that the effect is due to the ability to discriminate stress at the phonological level in the bilingual group dominant in the stress-contrasted language. One way to test this would be to conduct an experiment similar to [33] testing lists of phonetically varied words rather than just different tokens of a single item, with the prediction that they, unlike the monolingual infants tested in [33], would discriminate. Alternatively, the effect in the present bilingual group might be restricted to the phonetic level. If this were the case, it is possible that the level of sensitivity of a given bilingual might depend on the degree of overlap of the acoustic cues that mark lexical stress in the native language and in the language used for the test stimuli. This could be tested by adopting the strategy complementary to the one used here, namely by testing two groups of homogeneous French/other language bilinguals, choosing one language that marks stress very similarly and one very differently from the test stimuli, predicting better performance for the group learning the language with similar stress cues.

Turning now to the balanced bilinguals, no significant discrimination was found, which might attest that these infants have difficulties at processing stress contrast information, and might be reminiscent of the U-shaped curves found in some studies on the acquisition of segmental information [15], [17], [50]. However, there was a tendency to observe a novelty effect (present in 11 of the 16 infants tested, who heard 6 of the 9 languages heard by infants in this group) as found for the other subgroup of bilinguals, which raises the possibility of a more fragile effect on sensitivity to lexical stress even in this balanced group, albeit one that is not strong enough to lead to a clear discrimination effect in the present study. In both cases, we suspect that a significant effect might be revealed by a different task such as the anticipatory eye movement paradigm (see [18]–[19]. Moreover, as discussed above, none of the infants included in the present study was hearing German, the language in which the stimuli had been recorded, and this acoustic distance to native language input might have made the task more difficult for the infants. It is thus possible that balanced French-German bilinguals would perform above chance level in the present experiment, a possibility that would support an effect of acoustic proximity on the increase of stress pattern discrimination (and thus an effect at the phonetic level). Importantly for infants raised in a balanced bilingual environment, it would establish that such infants are able to discriminate prosodic contrasts present in one of their native languages, even if these contrasts are not present in their other language, extending previous research conducted on a similar issue for the acquisition of the phonemic categories.

At this point, we would like to discuss two related limitations to our study. The first one is that because the French-other language bilinguals had many different other languages, we do not know how the level of performance of the bilinguals compares to the performance of monolinguals of those same stress-contrasted languages. Therefore, even though we established that at least the bilinguals dominant in the stress-contrasted languages performed better than the French monolinguals, as we had predicted, we cannot ascertain that they perform at the same level as monolinguals of stress contrasted languages. Future research will have to evaluate this by testing homogeneous groups of bilinguals and their monolingual counterparts for both languages. The second limitation has to do with the fact that we only tested one age group, 10-month-olds, based on evidence that prosodic processing is already language-specific at this age in monolingual populations [30]–[31], [33]. However, given U-shaped trajectories observed for the acquisition of some phoneme categories [15], [50], future research should further explore prosodic acquisition from a developmental perspective, testing at different ages two monolingual populations (e.g. French- and German-learning infants) and a homogeneous population of bilinguals learning these two languages. Such a study would also shed light on one point that remains unanswered from the present study, namely the question of whether the better stress pattern discrimination ability of the bilinguals dominant in the stress-contrasted language as compared to French-learning monolinguals is due to decrease in performance in the French-learning monolinguals, increase in performance in the bilinguals, or a combination of both.

In summary, a growing body of research is attempting to understand how the bilingual environment affects the perceptual processing of phonological (segmental and prosodic) information in newborns and young infants [8], [14], [15], [17], [21], [51]. Our study is the first to contribute to this literature with respect to the processing of stress pattern at the lexical level.
